# Accurate Prediction of Protein Structural Class

**DOI:** 10.1371/journal.pone.0037653

**Published:** 2012-06-19

**Authors:** Xia-Yu Xia, Meng Ge, Zhi-Xin Wang, Xian-Ming Pan

**Affiliations:** Ministry of Education, The Key Laboratory of Bioinformatics, School of Life Sciences, Tsinghua University, Beijing, China; University of South Florida College of Medicine, United States of America

## Abstract

Because of the increasing gap between the data from sequencing and structural genomics, the accurate prediction of the structural class of a protein domain solely from the primary sequence has remained a challenging problem in structural biology. Traditional sequence-based predictors generally select several sequence features and then feed them directly into a classification program to identify the structural class. The current best sequence-based predictor achieved an overall accuracy of 74.1% when tested on a widely used, non-homologous benchmark dataset 25PDB. In the present work, we built a multiple linear regression (MLR) model to convert the 440-dimensional (440D) sequence feature vector extracted from the Position Specific Scoring Matrix (PSSM) of a protein domain to a 4-dimensinal (4D) structural feature vector, which could then be used to predict the four major structural classes. We performed 10-fold cross-validation and jackknife tests of the method on a large non-homologous dataset containing 8,244 domains distributed among the four major classes. The performance of our approach outperformed all of the existing sequence-based methods and had an overall accuracy of 83.1%, which is even higher than the results of those predicted secondary structure-based methods.

## Introduction

The tertiary structures of proteins with high molecular specificity are believed to play key roles in performing their biological functions. However, the increasing gap between the output of sequencing and structural genomics creates difficulty in the advancement of research. To obtain additional knowledge about proteins, scientists have focused on structural space, and two comprehensive databases, SCOP [1] and CATH [2], which describe protein structural and functional relationships using a hierarchy of classifications, have been constructed. These two methods categorize protein domains into classes based on the grouping of assigned folds, which are categorized according to the contents and spatial arrangements of the secondary structural elements of the protein domains [3]. The current version of the SCOP database, v. 1.75, includes eleven structural classes, with the four major classes (all-α, all-β, α/β and α+β) covering approximately 90% of the entries. Slightly different from SCOP, CATH does not differentiate between α/β and α+β domains at the class level (these are treated together as mixed αβ) but further classifies these domains into different topologies. The annotated protein domains are quite limited compared with the 13,116,724 non-redundant protein sequences in the NCBI RefSeq database (v. 48) [4], which increase the need for accurate and automated sequence-based protein structural class prediction methods. Correct prediction of protein structural classes has been proven useful for the prediction of protein secondary and tertiary structures [5], [6].

During the past three decades, many computational approaches have been developed for predicting the structural class of protein domains from their amino acid (AA) sequences. These approaches differ mainly in the features selected to represent the AA sequences and the classification algorithms. The early approaches were primarily based on the AA composition, and treated protein domains as 20-dimensional (20D) vectors corresponding to the frequencies of the twenty types of AAs [7]–[11], based on the discovery of Muska and Kim that the structural class of a protein domain correlates strongly with its AA composition [12]. After realizing that such approaches ignored information on the sequence order, which is also correlated with the protein structural class, the so-called *pseudo*-AA composition (PseAAC) [13], [14] and polypeptide composition [15], [16] were introduced to overcome the limitation. Several other features [17]–[25], such as the autocorrelation function based on the non-bonded residue energy [17], complexity measure factors [18], functional domain composition [19], and features extracted from the Position Specific Scoring Matrix (PSSM) [20], [21] and predicted secondary structure [3], [22]–[24] have also been applied to represent AA sequences. These selected features were then fed into various classification algorithms, such as fuzzy clustering [26], component-coupled [27], Bayesian classification [28], neural networks (NNs) [29], logistic regression [30], [31] and support vector machine (SVM) algorithms [14], [22], [23], [32], [33]. Without considering the predicted secondary structural information, such prediction methods achieved accuracies close to or greater than 90% when tested on datasets of limited size or relatively high sequence identity but performed poorly on datasets that were expanded or characterized by low, twilight-zone identity, with accuracies between 50 and 70% [3]. Considering that the structure of a protein is determined by its amino acid sequence [34], improvements in the sequence-based prediction methods are promising.

In the present work, we developed an approach that predicts domains into the four major SCOP classes (all-α, all-β, α/β and α+β) by converting each domain into a discriminating 4-dimensional (4D) structural feature vector solely based on the 440-dimensional (440D) sequence feature vector extracted from the PSSM. At first, each domain in the training set was assigned to an approximate 4D structural feature vector based on the composition of its secondary structural elements and to another 440D sequence feature vector based on its PSSM profile. Assuming that the domains’ 4D structural feature vectors were linear combinations of their 440D sequence feature vectors, the regression coefficient matrix was determined by using iterative least-squared multiple linear regression (MLR) method [35] based on the training data. Using the estimated coefficient matrix, the 4D structural vectors of the domains in the testing set were calculated according to their 440D sequence feature vectors, and then utilized to predict the four major classes. We employed 10-fold cross-validation and jackknife tests [36] to train and evaluate the model on a large, non-homologous dataset containing 8,244 domains selected from the ASTRAL SCOP40 v. 1.73 dataset [37], and an overall accuracy of 83.1% (jackknife test) was achieved. A blind test was also conducted on another dataset comprising 1,185 domains that are not included in SCOP v. 1.73 but are included in SCOP v. 1.75 to evaluate the unbiased performance of the method; an overall accuracy of 80.1% was achieved. The performance of our approach outperformed all of the existing sequence-based methods and was even better than those predicted secondary structure-based methods.

## Results

### Discriminative Ability of the Structural Feature Vectors

**Figure 1:**
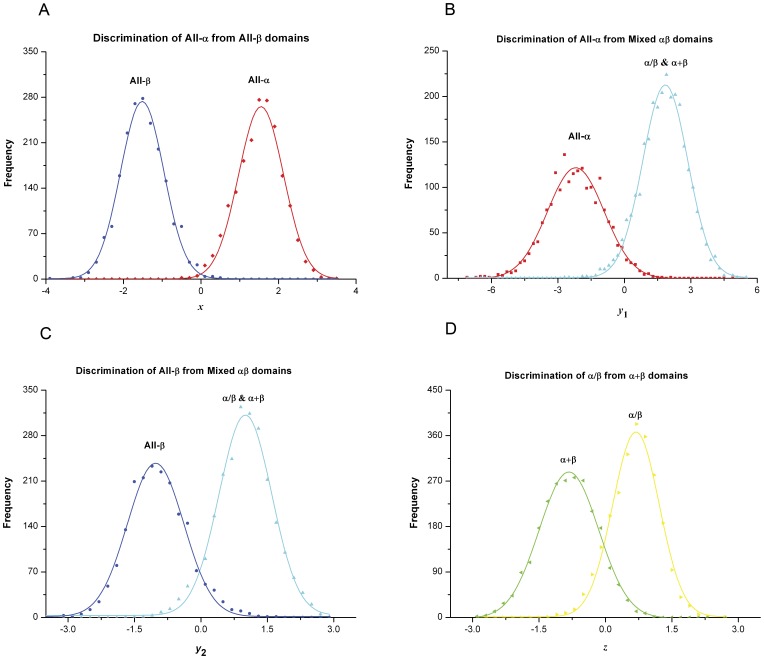
Discrimination of the protein domains between paired structural class groups. A) Discrimination of the all-α from all-β domains. B) Discrimination of the all-α from mixed αβ domains. C) Discrimination of the all-β from mixed αβ domains. D) Discrimination of the α/β from α+β domains.

To obtain the regression coefficient matrix *A_shaps,seq_*, which links the structure feature vector *V_shaps_* to the sequence feature vector *V_seq_*, the 8,244 domains were grouped into the following four subsets: 1) 3,673 all-α vs. all-β domains; 2) 6,315 all-α vs. mixed αβ (α/β and α+β) domains; 3) 6,480 all-β vs. mixed αβ domains; and 4) 4,571 α/β vs. α+β domains. 10-fold cross-validation was performed to train the MLR model and to test the discrimination ability of the calculated structural vectors. The 4D structural feature vectors of the domains in the training set were calculated according to Eq. 2, and the corresponding 440D sequence feature vectors were obtained from their PSSMs according to Eq. 3. Two rounds of least-squared MLRs were employed to determine the regression coefficient matrix *A_shaps,seq_*. Using the trained coefficient matrix, the 4D structural vectors were calculated from the 440D sequence vectors for each domain in the testing set. Within each of the four subsets, the scores of a certain dimension (*x, y*_1_, *y*_2_, *z*) of the domains’ 4D structural feature vectors can clearly differentiate between the corresponding two groups of domains. As shown in Figure 1, the discriminative accuracy was 99.5% for the first subset, containing the all-α vs. all-β domains, 95.6% for the second subset, containing the all-α vs. mixed αβ domains that have *x*>0, 92.7% for the third subset, containing the all-β vs. mixed αβ domains that have *x*<0, and 89.4% for the last subset, containing the α/β vs. α+β domains. The structural class of any query domain could be predicted by combining two of the four types of discrimination. More specificially, the structural feature scores *x*>0 and *y*_1_<0 determine an all-α domain, *x*<0 and *y*_2_<0 determine an all-β domain, *y*_1_>0 or *y*_2_>0, together with z>0, determine an α/β domain, and *y*_1_>0 or *y*_2_>0, together with z<0, determine an α+β domain.

### Structural Class Prediction Accuracies

The results of 10-fold cross-validation and jackknife tests performed on the D8244 dataset are summarized in Table 1. Based on the results, the overall accuracy of our method is high (83.1%), and the GC^2^ value [38] of our method achieved 0.56 using the dataset D8244. A blind test was also conducted on the independent D1185 dataset comprising 1,185 domains that are in SCOP v. 1.75 but not in v. 1.73 to evaluate the unbiased performance of the method. Based on the results shown in Table 2, a high overall accuracy (80.1%) was achieved.

**Table 1:** Performance of the 10-fold cross-validation and jackknife tests using the D8244 dataset.

Class	10-fold Cross-validation				Jackknife			
	Sn (%)	Sp (%)	MCC	GC^2^	Sn (%)	Sp (%)	MCC	GC^2^
All-α	91.9	97.2	0.88		92.0	97.3	0.89	
All-β	84.6	96.1	0.82		85.0	96.2	0.82	
α/β	83.1	94.4	0.78		83.2	94.5	0.79	
α+β	73.7	89.0	0.62		74.4	89.0	0.63	
Overall	82.8			0.56	83.1			0.56

**Table 2:** Performance of the blind test using the independent D1185 dataset.

Class	Accuracies			
	Sn (%)	Sp (%)	MCC	GC2
All-α	95.6	95.6	0.88	
All-β	81.0	94.7	0.76	
α/β	78.9	94.2	0.71	
α+β	71.9	87.4	0.60	
Overall	80.1			0.50

According to Table 1, the prediction of the all-α domains has the highest sensitivity, specificity, and MCC values among the four structural classes, indicating that the prediction of domains in this class is the most reliable. As shown in Table 2, this advantage is also reflected in the blind test using the D1185 dataset, in which only 11 of the 251 all-α domains (4.4%) were mispredicted. In contrast, the prediction of the α+β domains is inferior to that of the remaining three classes, suggesting that difficulty exists in recognizing the anti-parallel β sheets. This disadvantage is also reflected in the blind test (Table 2) in which 134 of the 477 α+β domains (28.1%) were mispredicted. Although many more α+β domains were involved in decreasing the overall prediction accuracy for the D1185 dataset to approximately 80.1%, the overall prediction accuracy is still much higher than that achieved by previous sequence-based algorithms.

### Comparison with Other Prediction Methods

**Figure 2:**
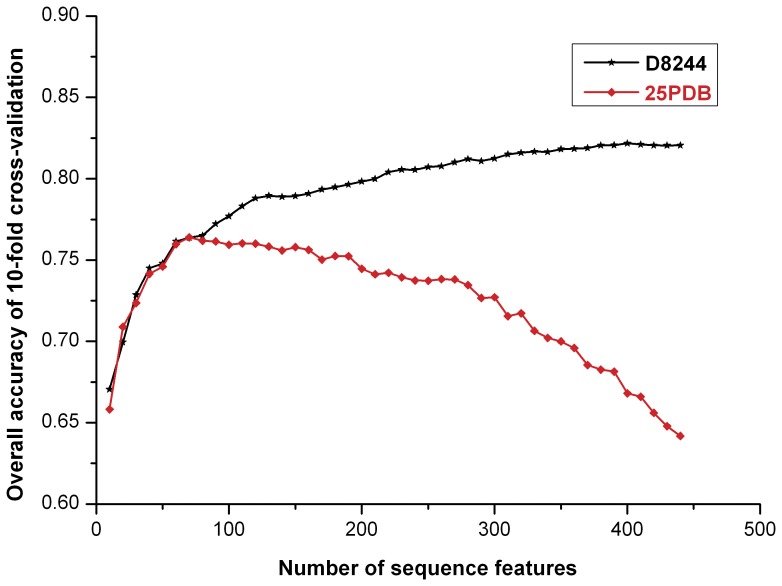
The effect of the number of sequence features used on the overall prediction accuracies for the two datasets 25PDB and D8244.

Because a widely used, low-identity dataset, 25PDB, is often used to evaluate the performance of protein structural class prediction methods [39], we also tested our method using the 25PDB dataset to compare side-to-side the performance of our approach with that of other methods. However, due to the limited size of 25PDB, the utilization of all 440 features would cause heavy over-fitting, which would seriously degrade the prediction accuracy. Figure 2 shows the relationship between the overall accuracy and the number of selected feature vectors. Using 25PDB, the overall prediction accuracy of 10-fold cross-validation first increased rapidly when less than 60 features were used, whereas the accuracy then increased more slowly until the highest overall accuracy of 77.0% was achieved with the utilization of 120 optimal features for each dimension; the accuracy gradually declined thereafter. When using D8244, the overall accuracy increased gradually still until all of the 440 feature vectors were included. These results suggest that, the limited number of features does not cover sufficient sequence information to characterize the structural information and that further sequence features are required to achieve a better result.

The jackknife test was performed using the 25PDB dataset, using 120 optimal features for each dimension. The performance of our approach versus other methods is shown in Table 3. Most of the previous sequence-based methods achieved an overall prediction accuracy of less than 70.0%, along with the GC^2^ value ranging from 0.06 to 0.28, highlighting the poor performance. In contrast, the overall prediction accuracy of our approach is high (up to 77.2%), which is close to the performance of the predicted secondary structure-based methods. This level of performance, achieved using only 120 features for each dimension, should be away from the expected performance of our method.

**Table 3:** Comparison of the jackknife test results between our method and other competing structural class prediction methods using the 25PDB dataset.

Algorithm	Reference	Accuracies					GC^2^
		All-α	All-β	α/β	α+β	Overall	
SVM (Gaussian kernel)	[33]	68.6	59.6	59.8	28.6	53.9	0.17
Bagging with random tree	[11]	58.7	47.0	35.5	24.7	41.8	0.06
Logistic regression	[30]	71.1	65.3	67.1	37.3	60.0	0.25
StackingC ensemble	[30]	74.6	67.9	70.2	32.4	61.3	0.26
Specific tri-peptides	[16]	60.6	60.7	67.9	44.3	58.6	–
LLSC-PRED	[31]	75.2	67.5	62.1	44.0	62.2	0.27
SVM	[31]	77.4	66.4	61.3	45.4	62.7	0.28
AAD-CGR	[25]	64.3	65.0	65.0	61.7	64.0	–
CWT-PCA-SVM	[14]	76.5	67.3	66.8	45.8	64.0	–
AADP-PSSM	[20]	83.3	78.1	76.3	54.4	72.9	–
AAC-PSSM-AC	[21]	85.3	81.7	73.7	55.3	74.1	–
SCPRED	[3]	92.6	80.1	74.0	71.0	79.7	0.55
MODAS	[22]	92.3	83.7	81.2	68.3	81.4	0.58
RKS-PPSC	[24]	92.8	83.3	85.8	70.1	82.9	–
SVM	[23]	92.6	81.3	81.5	76.0	82.9	–
This work		92.6	72.5	71.7	71.0	77.2	0.50

## Discussion

In the present work, we employed a MLR model to transform the 440D sequence feature vector extracted from the PSSM of a protein domain into a 4D structural feature vector, and, the structural class of the domain was then predicted. We performed 10-fold cross-validation and jackknife tests on the large non-redundant dataset D8244 to evaluate the performance of our method. A high overall accuracy of 83.1% (jackknife test) was achieved, which is even higher than that of those predicted secondary structure-based methods. A blind test was also performed using another dataset of updated domains, D1185, and the method provided an overall accuracy of 80.1%. Moreover, to compare the performance of our approach with that of the other methods, we also tested our approach on a widely used, low-identity dataset, 25PDB. Due to the limited size of the 25PDB dataset, the utilization of all 440 of the features would cause heavy over-fitting, which would seriously degrade the prediction accuracy. For this reason, we only used 120 optimal features for each dimension, which provided an overall prediction accuracy of 77.2%. Although the accuracy is away from the best performance of our approach, it is still higher than that of the existing sequence-based methods and is even close to that of those predicted secondary structure-based methods (Table 3). The improved performance of our methods is due to the effective utilization of sequence features and also to a bridging of the gap between the sequence and structural features that directly differentiate between the domains of the four classes. Furthermore, there is a limitation in the predicted secondary structure based methods; their prediction accuracies rely heavily on the accuracies of the underlying secondary structure prediction methods. Considering that current secondary structure prediction methods achieve an average accuracy close to 80.0% [3], it would be difficult for structural class prediction methods based on them to improve much farther. Moreover, our method directly maps each domain into a 4D structural space based solely on the PSSM, which bridges the gap between the protein sequence space and the structural space and provides further research possibilities regarding the protein sequence-structure-function relationships.

## Materials and Methods

### Datasets

#### D8244

To train and test the model, a large dataset containing 8,244 domains distributed among the four major classes (all-α, all-β, α/β and α+β) was constructed as described below. A subset containing domains with pair-wise sequence identities of no more than 40% was downloaded from the ASTRAL compendium (v. 1.73) [37]. Domains that are either discontinuous, have a sequence length of less than 30 residues or with limited number of residues resolved (<50%), or belonging to classes other than the four major classes were removed. The final dataset, named D8244, comprises 8,244 domains that are located in 6,775 protein sequences. Of these 8,224 domains, 1,744 belong to the all-α class, 1,929 belong to the all-β class, 2,357 belong to the α/β class, and the remaining 2,214 belong to the α+β class. The PSSM, which provides the evolutionary information was generated for each of the 6775 protein sequences by searching against the NR database (v2.2.1, downloaded on Jul 8, 2011) using Position-Specific Iterated BLAST (PSI-BLAST) (-j 3–h 0.001) [40]. Detailed information of the D8244 dataset is shown in supplementary table S1.

#### D1185

Another blind test set containing 1,185 domains was constructed to evaluate the unbiased performance of the method. All of the domains that were updated between SCOP v. 1.73 and SCOP v. 1.75 were downloaded. Similarly, only continuous domains with a sequence length of more than 30 residues and belonging to the major four classes were retained. These domains were filtered using a clustering program CD-HIT [41] at a 40% sequence identity, and the remaining domains with more than 40% identity with any domain in D8244, according to the CD-HIT-2d [41] were removed. This final blind dataset, named D1185, includes 1,185 domains from 1,023 protein sequences. Of these 1,185 domains, 251 belong to the all-α class, 258 belong to the all-β class, 199 belong to the α/β class, and the remaining 477 belong to the α+β class. PSSMs were obtained for all 1,023 of the sequences as described above. Detailed information of the D1185 dataset is shown in supplementary table S2.

#### 25PDB

The 25PDB dataset was originally constructed by Kurgan and Homaeian [39] and has since been widely used as a benchmark dataset by other researchers. In the present work, this dataset was employed to test the current method and facilitate its comparison with other methods. Domains in 25PDB were selected from high-resolution protein structures, with low pairwise sequence identity (no more than 25%). The 1,673 domains include 443 all-α, 443 all-β, 346 α/β, and 441 α+β domains obtained from 1,527 protein sequences. The PSSMs for all 1,527 of the protein sequences were obtained as described above.

### Composition and Distribution of the Secondary Structure Elements

A function describing the composition of the secondary structure elements along the backbone of each protein domain was constructed. For a given protein domain with a sequence length *L*, if the number of secondary structure elements in a segment of length *k* is {*n_i_*} (*i*∈{α-helix, anti-parallel β sheet, and parallel β sheet}), then the composition of these three elements in the segment should be {*n_i_/k*}. “Walking” along the domain sequentially, a universal function that calculates the average composition of the three secondary structure elements in all segments is expressed by the following equation:
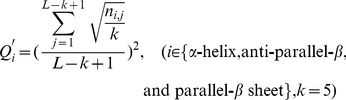
(1)


In this equation, *n_i,j_* is the number of the *i*th element in a segment of length *k* around position *j* and, consequently, 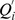 describes the spatial organization of the secondary structure elements of a protein domain. If a certain type of secondary structure element *i* is uniformly distributed along the protein sequence, then 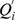 should be equal to the content of this secondary structure element calculated using the entire sequence 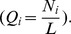


### 4D Structural Feature Vector

According to SCOP’s definition of structural classes, the domains in various structural classes are differentiated in their composition and in the arrangements of their secondary structure elements. In this study, a 4D structure feature vector *V_shaps_* reflecting these differences was constructed based on the corresponding value of 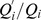 for each domain. Revisions to these 4D vectors were conducted to ensure that the domains of different structural classes are located in different regions of the 4D structural space. Each of the four dimensions differentiates between the domains of two classes, as follows: the first feature score (named *x*) primarily differentiates all-α from all-β domains:
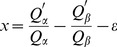
(2a)


In this equation, *ε* is the structural class-related variable whose initial value was arbitrarily assigned to ensure that the *x* scores of the all-α domains are greater than zero and those of the all-β domains are less than zero.

The second feature score (named *y*_1_) differentiates all-α from mixed αβ (α/β and α+β) domains that have *x* scores greater than zero:
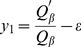
(2b)


As above, the initial value of *ε* was assigned to ensure that the *y*_1_ scores of the all-α domains are less than zero and those of the mixed αβ domains are greater than zero.

The third feature score (named *y*_2_) differentiates all-β from mixed αβ domains that have *x* scores less than zero:
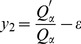
(2c)


As above, the initial value of *ε* was assigned to ensure that the *y*_2_ scores of the all-β domains are less than zero and those of the mixed αβ domains are greater than zero.

The fourth feature score (named *z*) differentiates α/β from α+β domains as follows:
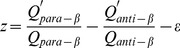
(2d)


Following the above description, the initial value of *ε* was assigned to ensure that the *z* scores of the α/β domains are greater than zero and those of the α+β domains are less than zero.

### 440D Sequence Feature Vectors

Another 440D sequence feature vector *V_seq_* was also extracted from the PSSM for each domain. The PSSMs generated using PSI-BLAST are powerful resources for constructing feature sets and have been widely used in bio-computational prediction tools. The evolutionary information summarized in PSSMs generalizes the attribute of each position in the protein sequence, ultimately improving the sensitivity of the prediction model. For a query domain with a sequence length of *L*, the PSSM is an L*20-dimensional score matrix, *P_i,j_* (*i* = 1,2,…,*L*; *j* = 1,2,…,20). The (*i*,*j*)th entry of the profile is a nominal score that represents the occurrence of the AA in position *i* of the query domain sequence that has been substituted by an AA of type *j* during evolution. The value of *P_i,j_>0* indicates that the occurrence of the amino acid in position *i* substituted by the amino acid type *j* is more frequent than that of the *pseudo*-count; otherwise, the occurrence of this substitution is less frequent. In the present work, we separately considered the AA composition of the domains, autocorrelations between residues, and several other variables related to the residue’s position in the sequence. The details of the 440 features are the following:

1) 20 features measuring the square root of the AA composition, with *N_j_* denoting the occurrence of AA type *j* appearing in the query sequence:

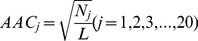
(3a)2) 40 features measuring the average score of the AAs in the query domain being mutated to AA type *j* during the evolutionary process, with 20 for *P_i,j_>0* :
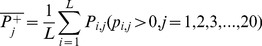
(3b)and the remaining 20 for Pi,j<0 :

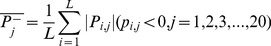
(3b́)3) 20 features measuring the autocorrelation of the hydrophobicity index of two residues separated by a distance of *k*, with 10 for *P_i,j_>0* :
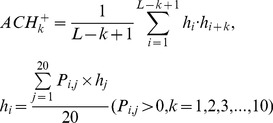
(3c)and the remaining 10 for Pi,j<0 :

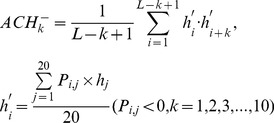
(3ć)4) 20 features measuring the autocorrelation of the side chain masses of two residues separated by a distance of *k*, with 10 for *P_i,j_>0* :
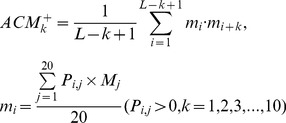
(3d)and the remaining 10 for Pi,j<0 :

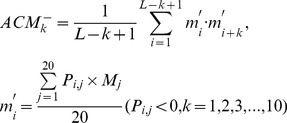
(3d́)5) 20*7 features measuring the autocorrelation of the PSSM scores of two residues separated by a distance of *k*:

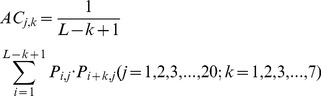
(3e)6) 40*3 features measuring the square value of the average score for segments of length *k* along the backbone, with 20*3 for *P_i,j_>0* :
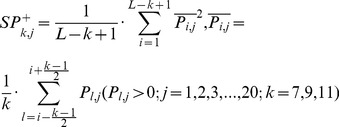
(3f)and the remaining 20*3 for Pi,j<0 :

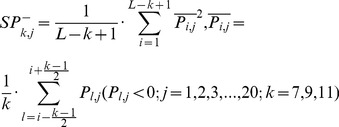
(3f́)7) 20*4 features measuring the mutation position preference of AA type *j*:
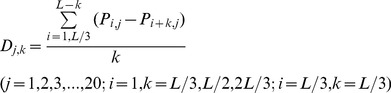
(3g)

### Iterative multiple linear regression (MLR)

Assuming that the structural vector *V_shaps_* are a linear combination of those of the sequence vector *V_seq_*, then the following equation holds:



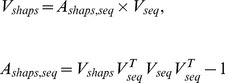
(4)


In this equation, *A_shaps,seq_* is a 4×441 coefficient matrix linking *V_shaps_* to *V_seq_*, and consequently, 1764 coefficients require estimation. Using the 4D structural feature vectors and the 440D sequence feature vectors of the domains in the training set, all of the coefficients can be estimated using the MLR method to minimize the sum of the squares of the deviations between the left- and right-hand sides of Eq. 4, as previously described [35]. In the present work, a two-step iterative MLR procedure was employed to optimize the coefficient matrix. Once the coefficient matrix is obtained, the 4D structural feature vector for any structure-unknown domain can be calculated from the PSSM of its AA sequence, and its structural class can then be predicted.

### Sequential Forward Stepwise Regression

When testing the method on a dataset of limited size, over-fitting becomes a problem and will heavily affect the prediction accuracy. In the present work, we used a simple sequential forward stepwise regression method to search for the optimal group of features for such datasets. For each dimension of the structure vectors, the sequence feature that correlated most strongly with it was chosen first. We note that the chosen sequence features for the four dimensions of the structure vectors need not be the same. Sequentially, the remaining feature that performed the best with the combined chosen features was added to the MLR model, until all of the features were included.

### Performance Measures

In the present work, 10-fold cross-validation and jackknife tests [36] were employed to evaluate the performance of the 4D structural feature vectors over the large D8244 dataset selected from SCOP40 v. 1.73. Furthermore, a blind test was also performed on another independent dataset, D1185, containing low-identity domains that were updated between SCOP v. 1.73 and SCOP v. 1.75 to assess the unbiased prediction performance. To evaluate the performance comprehensively, the standard prediction accuracies and Matthews correlation coefficients (MCC) over each of the four structural classes were reported, as were the overall accuracy and the generalized squared correlation (GC^2^) over the entire dataset. Both the MCC and GC^2^ are related to χ^2^ statistics [38]. The MCC is used to measure the quality of binary classifications, and returns a value ranging between −1 and 1, with 0 representing random correlation, and greater positive (negative) values indicating a higher (lower) prediction quality for a given class. When there are more than two classes for prediction, the GC^2^ is required instead, and its value ranges between 0 and 1 in which, 0 corresponds to the worst classification (no correct predictions) and 1 corresponds to a perfect classification [38]. These parameters are detailed in the following equations:



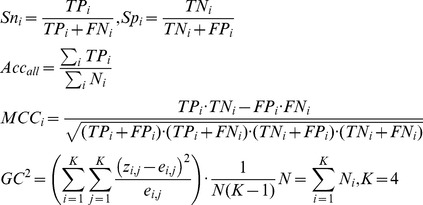
(5)


## Supporting Information

Table S1 List of the D8244 dataset.
(XLS)

Table S2 List of the D1185 dataset.
(XLS)
